# Clinging to the top: natal dispersal tracks climate gradient in a trailing-edge population of a migratory songbird

**DOI:** 10.1186/s40462-024-00470-0

**Published:** 2024-04-16

**Authors:** Heather E. Gaya, Robert J. Cooper, Clayton D. Delancey, Jeffrey Hepinstall-Cymerman, Elizabeth A. Kurimo-Beechuk, William B. Lewis, Samuel A. Merker, Richard B. Chandler

**Affiliations:** 1 Warnell School of Forestry and Natural Resources, University of Georgia, 180 E Green Street, Athens, GA 30602 USA; 2 Southeastern Cooperative Wildlife Disease Study, College of Veterinary Medicine, University of Georgia, 589 D. W. Brooks Drive, Athens, GA 30602 USA; 3 Department of Ecology and Evolutionary Biology, University of Connecticut, 75 N. Eagleville Road, Storrs, CT 06269 USA

**Keywords:** Climate gradient, Hierarchical model, Migration, Movement model, Natal dispersal, Neotropical migrants, Trailing-edge

## Abstract

**Purpose:**

Trailing-edge populations at the low-latitude, receding edge of a shifting range face high extinction risk from climate change unless they are able to track optimal environmental conditions through dispersal.

**Methods:**

We fit dispersal models to the locations of 3165 individually-marked black-throated blue warblers (*Setophaga caerulescens*) in the southern Appalachian Mountains in North Carolina, USA from 2002 to 2023. Black-throated blue warbler breeding abundance in this population has remained relatively stable at colder and wetter areas at higher elevations but has declined at warmer and drier areas at lower elevations.

**Results:**

Median dispersal distance of young warblers was 917 m (range 23–3200 m), and dispersal tended to be directed away from warm and dry locations. In contrast, adults exhibited strong site fidelity between breeding seasons and rarely dispersed more than 100 m (range 10–1300 m). Consequently, adult dispersal kernels were much more compact and symmetric than natal dispersal kernels, suggesting adult dispersal is unlikely a driving force of declines in this population.

**Conclusion:**

Our findings suggest that directional natal dispersal may mitigate fitness costs for trailing-edge populations by allowing individuals to track changing climate and avoid warming conditions at warm-edge range boundaries.

## Background

Theoretical models of spatial population dynamics suggest that the effects of climate change on population viability and gene flow will depend on dispersal capacity [7, 36, 61]. Species that cannot track optimal climatic conditions via dispersal are likely to experience increased extinction risk from reductions in survival and reproduction [28, 47, 55]. However, studying dispersal in natural populations is notoriously difficult, especially for mobile species, and few empirical studies have investigated the degree to which dispersal is directed towards optimal climate conditions [51, 65].

Populations at the trailing edge of a shifting range provide many opportunities for investigating dispersal responses to climate change. Trailing-edge populations are often near their physiological thresholds [15, 23] and are therefore likely to be sensitive to novel abiotic conditions [6, 47, 60]. Suitable habitat is often more fragmented at the trailing edge than at the core of the range, constraining the available area for dispersal [20, 23, 24]. As a result, dispersal capacity is especially critical for trailing-edge populations facing climate-based extinction [27].

Dispersal capacity can vary with age, sex and other individual traits [22, 43]. Natal dispersal is typically greater than adult dispersal within vertebrates [17, 22, 50], suggesting that natal dispersal could be a key driver of climate-induced range shifts [10, 22, 49]. For adults, site fidelity offers numerous advantages such as increased mating success [25] and familiarity with available food resources [5, 21], whereas dispersal represents a risky trade-off (Fleischer et al, [16]; Bonte et al,[4], but see [45]). For young individuals, dispersal serves as a mechanism to seek higher-quality habitat [4, 9], avoid competition or inbreeding with relatives [9, 35], escape parasites [62], and increase mate availability [8, 26].

Migratory species face unique challenges from climate change, which can impact phenology, physiology, and demography at non-breeding and breeding sites throughout the annual cycle [46, 48, 56, 69, 71]. Studying these effects is complicated because changes in either survival or breeding site selection can make it difficult to observe dispersal events [44, 70]. Long-term studies of marked individuals occurring over strong climate gradients provide one of the few options for advancing knowledge of climate change impacts on demography and dispersal [11, 29].

We used 21 years of mark-recapture data from a trailing-edge population of black-throated blue warblers (*Setophaga caerulescens*) to test the hypothesis that a key mechanism underlying climate-induced range shifts of trailing-edge populations is directional (non-random) dispersal. For black-throated blue warblers, cooler and wetter conditions, which are positively correlated with elevation, provide the best breeding habitat [31]. At the trailing edge of the range, abundance has remained relatively stable at colder and wetter areas at higher elevations but populations have become extirpated at the warmer, drier sites at lower elevations [41, 57]. To evaluate the hypothesis that directional dispersal explains recent local range shifts, we tested the prediction that birds would be more likely to disperse to colder and wetter conditions in the surrounding landscape than to warmer and drier conditions. For young birds, dispersal decisions are likely to be influenced by the available environmental conditions relative to the hatch location. At the coldest and wettest sites, there are no available locations within the study area with better conditions. In contrast, a bird hatched at a lower elevation in warmer and drier conditions could have many available locations in the study area with better conditions. We further predicted that natal dispersal would be greater than adult dispersal because adults are known to exhibit high site fidelity [9].

## Methods

The black-throated blue warbler is a Neotropical migratory bird that winters in the Caribbean and Central America and breeds in the eastern United States and southeastern Canada. The southernmost breeding populations occur in the southern Appalachian Mountains. Black-throated blue warblers have been heavily studied in the core of their breeding range [9, 30], but less is known about trailing-edge populations [12].

In the core of the range, adult black-throated blue warblers exhibit strong site fidelity during the breeding season, and dispersal is influenced by both habitat structure and sex [9, 64]. Adult black-throated blue warblers rarely disperse from their chosen patch except in response to habitat disturbance [2, 3]. As with most Neotropical migratory songbirds, little is known about natal dispersal, but natal dispersal distances >1 km are thought to be common in the core of the range [31].

### Field methods

**Fig. 1:**
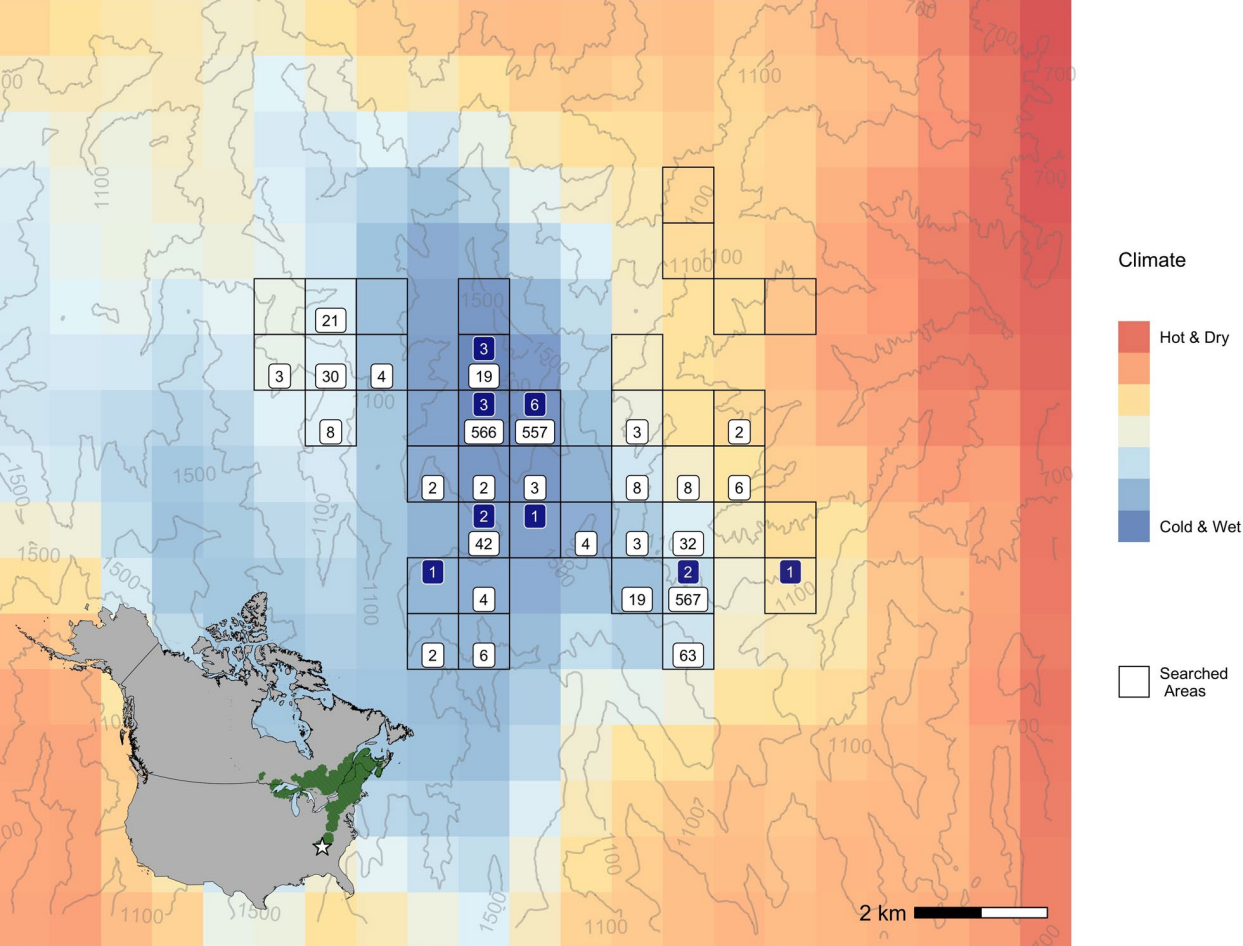
Locations of black-throated blue warblers banded as nestlings in the Nantahala National Forest, North Carolina, USA. From 2002 to 2022, 2072 nestling black-throated blue warblers were banded and released, of which 19 were recaptured as second-year birds. White squares represent the total number of nestlings banded in each grid cell. Blue boxes show the number of nestlings that were recaptured as second-year birds in that grid cell. Nestlings recaptured as after-second-year birds are not included in the figure. Some study plots span more than one grid cell. The background color depicts climate, represented as the dominant principal component of precipitation and temperature normals. Contour lines depict elevation in meters.The inset shows the breeding range of the black-throated blue warbler in green, with a star marking the study site location

From 2002 to 2022, we monitored black-throated blue warblers in the Nantahala National Forest in western North Carolina (35.1°N, 83.4°W), at the trailing edge of the range (Fig. 1). The southern Appalachian Mountains are characterized by steep topography ranging from 500 to 1600 m above sea level. Historically, both eastern hemlock (*Tsuga canadensis*) and American Chestnut (*Castanea dentata*) were common, especially in riparian areas [14]. The study site is now composed of mixed oaks (*Quercus* spp.), tulip poplar (*Liriodendron tulipifera*), hickories (*Carya* spp.), and maples (*Acer* spp.). Yellow birch (*Betula alleghaniensis*), black birch (*Betula lenta*), black cherry (*Prunus serotina*) and black gum (*Nyssa sylvatica*) are also common throughout the area. Understory foliage is dominated by rhododendron (*Rhododendron maximum*) and mountain laurel (*Kalmia latifolia*), with some dry sites lacking any shrub or mid-canopy layer. Temperature and precipitation are highly correlated with elevation. The coldest and wettest sites are found at the highest elevations. Mean annual precipitation increases from 1868 mm year^−1^ at 600 ms to 2514 mm year^−1^ at 1400 ms above sea level. Mean May air temperatures decrease from 17.5 °C at the lowest elevations to 13.9 °C at the highest elevations [13]. Caterpillar biomass (the primary food source for black-throated blue warblers during the breeding season [54]) is positively correlated with cooler climates at these sites [40].

We surveyed 19 study plots (each plot covering a $\sim12$ ha area) ranging in elevation from 600 m to 1500 m above sea level (Fig. 1). The study began with one lower elevation intensively-surveyed (henceforth ‘intensive’) plot in 2002, with a second higher elevation intensive plot added in 2003 and 17 auxiliary plots added between 2014 and 2018. More details on the starting date for each plot can be found in the data archive associated with this manuscript. Each intensive plot was surveyed approximately every 2 days during the breeding season to map black-throated blue warbler territories, assess breeding density, find nests, and monitor nesting success. Once found, nests were monitored until failure or successful fledging of chicks. All surviving nestlings were banded with USGS aluminum leg bands 6 days after hatching. Nestlings were not sexed during initial banding.

We attempted to capture, sex, and age all adult black-throated blue warblers on our intensive plots every year through targeted and constant-effort mist-netting. At the 17 auxiliary plots, we performed constant effort mist-netting for 4 days during the breeding season to band birds and monitor nests found during banding operations. In some years, nest searching and re-sighting surveys were also performed at some of these plots. Nest searching on these auxiliary plots was not exhaustive. All captured adult individuals were banded with a U.S. Geological Survey (USGS) aluminum leg band and a unique combination of colored leg-bands.

We used PRISM’s 30-year climate normals (1991–2010) [13] at an 800 m resolution to describe average May precipitation and temperature across our study area. After standardization, we conducted a principal component analysis [32] to create a single variable that represented the dominant climate gradient of the region. Higher values of this variable represented hotter and drier locations, with lower values representing wetter and colder sites.

### Modeling framework

We analyzed dispersal events of individuals captured between 2002 and 2022. We used a discrete-time, discrete-space extension of the Cormack–Jolly–Seber (CJS) [38, 58] model to draw inferences about dispersal. This hierarchical model has a state process describing the dispersal and survival of all individuals in the sample, not just the ones that were detected in subsequent years. The state process is treated as partially observed, with an observation process that accounts for imperfect detection and the fact that dispersal events can only be observed at sampling locations. The observation process is critical to avoid selection bias. Specifically, ignoring birds that were not recaptured would equate to conditioning on birds that survived and dispersed to our sampling locations. Since sampling was not uniform throughout the study area, this would result in bias, as it would incorrectly suggest that birds prefer to disperse to our study plots. By modeling all birds, and acknowledging that some birds died and moved to areas that we did not sample, we avoided this form of selection bias.

We divided the study area into $N = 320$ grid cells (Fig. 1) with each cell covering a 800 × 800 m area and snapped all locations to grid cell centers. Following the framework of Schick et al. [59], we defined ${\varvec{z}}_{i,t}$ as a vector of length *N*, indicating the location of individual *i* in year *t*. In other words, the vector ${\varvec{z}}_{i,t}$ contains zeros and a single one corresponding to the location where the bird occurred in year *t*. The location vector ${\varvec{z}}_{i,t}$ was modeled as an outcome of a multinomial distribution, conditional on the individual’s location and age (nestling or adult), *a*, in the previous year.

$$\begin{aligned} {\varvec{z}}_{i,t,a}|{\varvec{z}}_{i,t-1,a-1} \sim \textrm{Multinom}(1, {\varvec{\theta }}_{i,t,a}) \end{aligned}$$

The probability $\theta _{i,k,t,a}$ of dispersing to location *k*, conditional on surviving and returning to the study area, is calculated by normalizing the dispersal kernel:

$$\begin{aligned} \theta _{i,k,t,a} = \frac{h_{i,k,t,a}}{\sum _{k'=1}^{N} h_{i,k',t,a}} \end{aligned}$$

The dispersal kernel describes the relative probability that individual *i* with age *a* selects location *k* in year *t* given the available climate conditions and the distances from the origin at location *j*.

$$\begin{aligned} h_{i,k,t,a} = e^{-\beta _{1,a} X_{i,k} + \beta _{2,a} c_{k} } \end{aligned}$$

The variable $X_{i,k}$ is the distance in kilometers between the individual’s location at time $t-1$ and location *k*, and $c_{k}$ is the climate at location *k*. The *a* subscripts on the effects of distance and climate indicate that the effects were age-specific, which is equivalent to modeling an interaction between age and the two covariates. For black-throated blue warblers, we modeled movements between years as dependent on the precipitation and temperature at location *k*, combined into a single standardized climate variable.

This model acknowledges that the shape of the dispersal kernel can depend on location because climate varies spatially. The parameter $\beta_1$ is strictly positive, and it describes the effect of distance on dispersal. Large values of $\beta _1$ indicate short dispersal distances, small values describe a diffuse dispersal kernel. If there is no effect of climate ($\beta _2$ = 0), then the dispersal kernel is isotropic and an individual is predicted to follow a simple random walk. On the other hand, as $\beta _2$ decreases, the dispersal kernel will be skewed away from regions with greater values of *c* (warmer and drier conditions). In these cases, the model also acknowledges that an individual is less likely to disperse far if it was born in a location with optimal climate conditions. In other words, the distance and directionality of dispersal will depend on the available climate conditions in the surrounding landscape (e.g. all other grid cells in the study area).

To account for the uneven spatial and temporal distribution of survey effort across our study area, we modeled the detection of an individual, $y_{i,k,t}$, as a Bernoulli outcome with detection probability $p_k$. To differentiate between intensive and auxiliary plots, we assumed detection was dependent on plot type. To account for variation in effort, we included a binary variable, $s_{k,t}$, that represented if grid cell *k* was sampled in year *t*.

$$\begin{aligned}&p_k = {\left\{ \begin{array}{ll} p_1 s_{k,t} &{} \quad \text {if intensive plot} \\ p_2 s_{k,t} &{} \quad \text {if auxiliary plot} \\ \end{array}\right. } \\ \end{aligned}$$

For $p_1$, we used a Uniform(0.8, 1.0) prior, as we expected detection was close to 1 in these plots. For $p_2$, we used a LogitNormal (0, 1.78), which approximated a uniform distribution on the logit scale. We used Normal (0, 1) priors for $\beta _1$ and $\beta _2$.

Modeling all birds in the sample and not just the ones that were recaptured required that we account for survival. However, as in most mark-recapture studies, we cannot distinguish between permanent emigration out of the study area (defined here by the spatial region shown in Fig. 1) and mortality. We therefore modeled “apparent survival” ($\phi _a$) for each age group *a*, defined as the probability of surviving and remaining in the study area. We conditioned detection on apparent survival using a discrete-time Bernoulli model,

$$\begin{aligned} w_{i,t} \sim \textrm{Bern}(\phi _a w_{i,t-1}) \\ y_{i,k,t} \sim \textrm{Bern}(p_k w_{i,t}) \end{aligned}$$

where $w_{i,t}$ indicates if individual *i* was alive and in the study area in year *t*. We used a Uniform (0, 0.3) prior for nestlings survival and a Beta(5,2) prior for survival of adults to loosely restrict survival estimates to ranges reported in the literature.

We analyzed data from natal and adult birds together. Because we were unable to distinguish long-distance dispersal (i.e., dispersal beyond our study area) from mortality, our dispersal model was focused on short-distance dispersal. We therefore put priors on the dispersal distance parameter $\beta _{1,a}$ to constrain inference to dispersal within the study area. For this, we used an exponential distribution, $\beta _{1,a} \sim \textrm{Exponential}(0.1)$, which puts $<0.05$ prior probability on dispersal distances $>30 \;{\text{km}}$.

Analysis was performed in JAGS via the ‘rjags’ package in Program R [52, 53]. Convergence was assessed using visual inspection of three chains and the Gelman-Rubin statistic (r-hat <1.1) [18]. Each Markov chain was run for 15,000 iterations, resulting in 45,000 posterior samples.

## Results

**Fig. 2:**
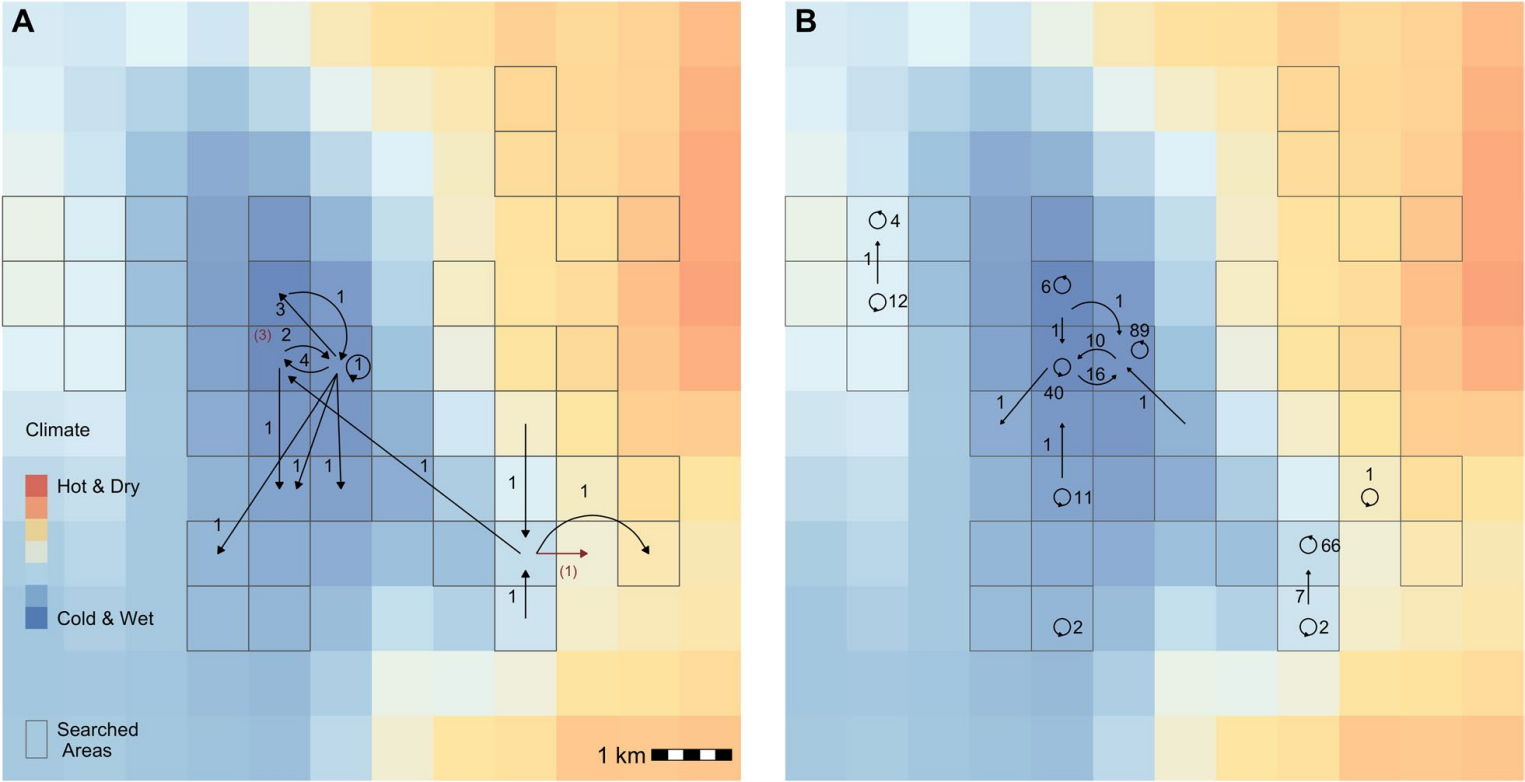
Dispersal events for black-throated blue warblers between consecutive years for **A** 23 nestlings and **B** 190 adult birds in the Nantahala National Forest, North Carolina, USA from 2002 to 2022. Arrows depict the number of individuals that dispersed between locations. Red arrows and numbers in **A** shows nestlings encountered more than one year after hatching. The background color depicts climate, represented as the dominant principal component of precipitation and temperature normals

We banded 2072 nestling black-throated blue warblers from 2002 to 2022. Of the nestlings banded, 24 were re-captured in the study area (1.2% return rate)—19 as first-time breeders (second-year birds, SYs) and 5 as adults (after-second-year birds, ASYs). Recaptured birds were male biased, with 16 males and 8 females recaptured. The median distance between nest location and first-year location was 917 m (range 23–3200 m) (Fig. 2). Median dispersal distance was farther for females (1092 m, range 356–3200 m) than males (812 m, range 235–2800 m). The majority (18 of 24) of the recaptured nestlings were born at the coldest and wettest sites above 1300 m elevation, where black-throated blue warbler density was highest. Two of the recaptured nestlings were born in the same nest, but established first-year territories 800 m apart. Yearly apparent survival, conditional on returning to the study area, was estimated to be 10% (6–14%) for yearlings.

We banded 1093 adult black-throated blue warblers (451 females, 632 males, and 10 with sex not recorded). We recaptured 190 individuals (114 males, 76 females) in subsequent years (17.4% return rate). Most recaptured birds were observed in two years, which were not always consecutive. One individual was observed in seven years. The median distance between territories in consecutive years was 79 m (range 10–1300 m) for adults, with females moving a median distance of 112 m (range 10–1300 m) compared to 71 m (range 10–1300 m) for males. Only 5 adults (3 SY females, 1 SY male, and 1 ASY male) were recorded moving > 500 m between years. Yearly apparent survival, conditional on returning to the study area, was 55% (50–61 %) for adult birds.

**Fig. 3:**
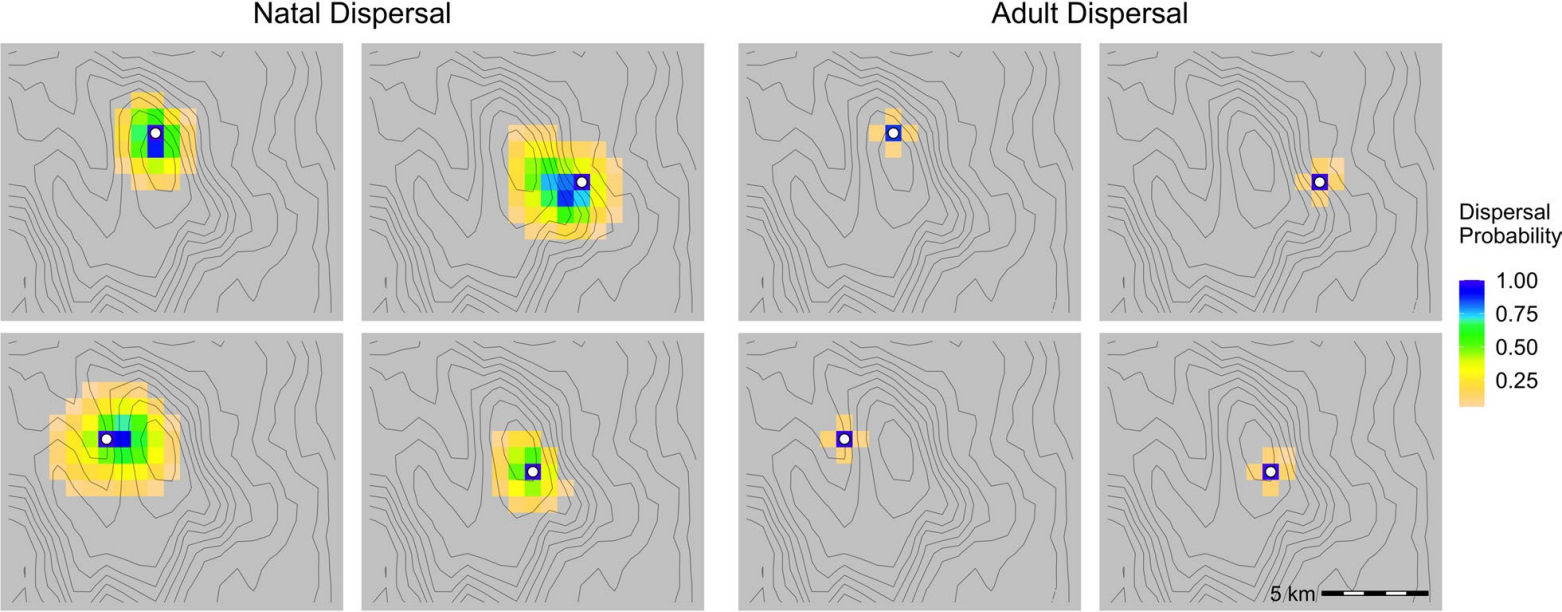
Predicted dispersal kernels of black-throated blue warblers at 4 theoretical nesting locations in the Nantahala National Forest, North Carolina, USA. Locations were chosen to represent dispersal patterns at 4 different climate conditions where black-throated blue warblers are present. White circles represent origin of dispersal. Grid cell colors represent the probability of dispersing to a location from the origin. Climate contours are shown as grey lines

**Fig. 4:**
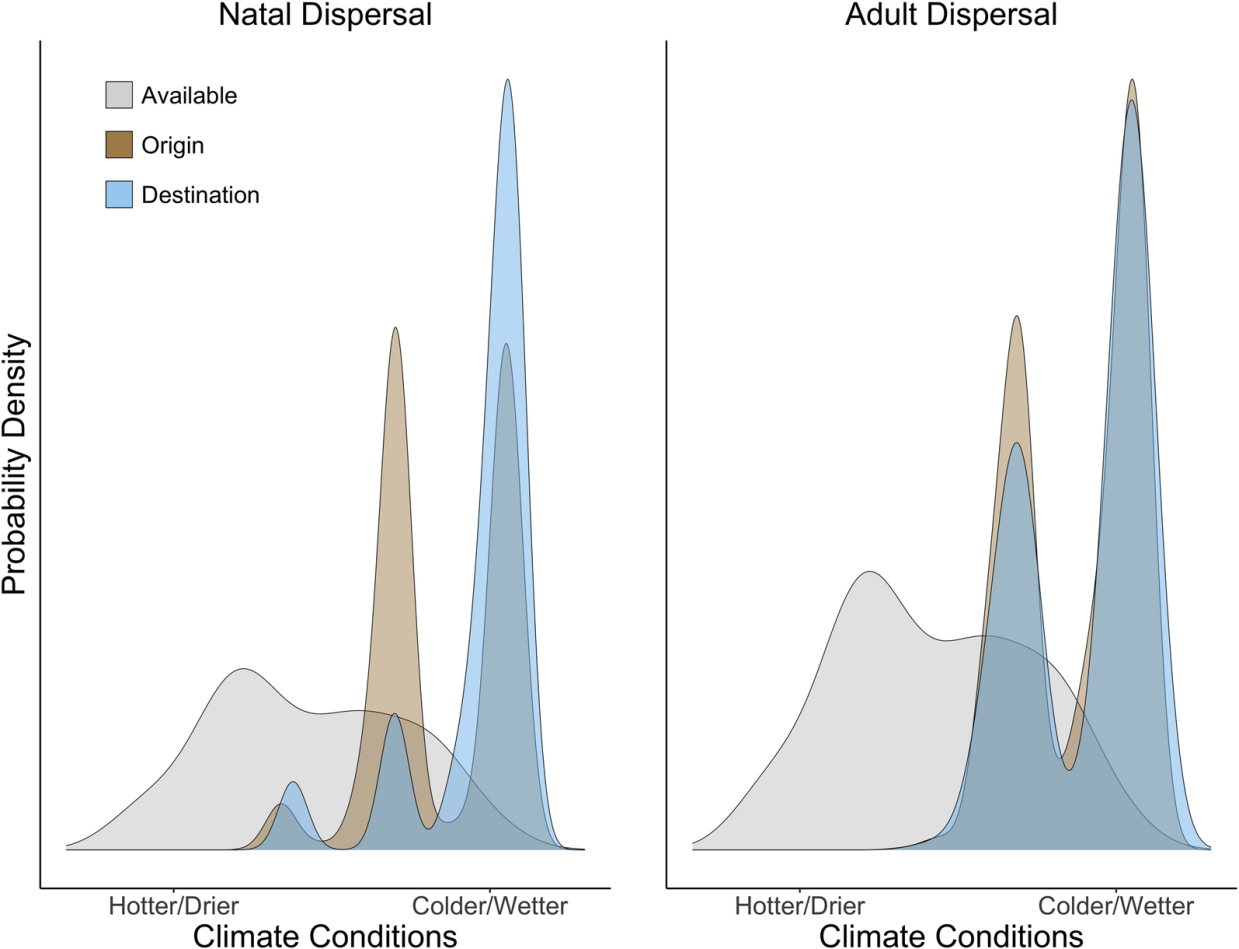
Climate conditions at dispersal destinations (blue distributions) compared to available climate conditions (all climate conditions present in the study area, grey distribution) for dispersal events of black-throated blue warblers banded in the Nantahala National Forest, North Carolina, USA. Conditions where black-throated blues were first banded at any age (nestlings or adults) is shown in brown. Nestlings recaptured more than one year after hatching are excluded from the nestling destination distribution

The effect of climate ($\beta _2$) on natal dispersal was $-$0.41, with a 95% credible interval that did not include zero ($-$0.74–$-$0.13), indicating that nestlings were more likely to disperse towards cooler and wetter locations relative to available conditions surrounding the nest sites (Fig. 3). Natal dispersal distances were shortest for individuals born at the highest elevations. For these individuals, there were no cooler climates available within the study area. This is consistent with the fact that no nestlings were observed to move to the low elevation sites with warmer, drier climates (Fig. 4). In contrast, natal dispersal distances were greater, and more directional, for nestlings hatched in the warmer, drier conditions at lower elevations.

Adult dispersal was more restricted and less directional than natal dispersal. Average adult dispersal distance was 79 m, compared to 917 m for recaptured nestlings. The estimated effect of climate on adult dispersal ($\beta _2$) was 0.18 with a 95% credible interval including zero ($-$0.06–0.54). Consequently, adult dispersal kernels were much more compact and symmetric than natal dispersal kernels (Fig. 3).

## Discussion

Trailing-edge range shifts can result from reduced vital rates or directional dispersal. Although both processes can cause local population declines, reduced survival and reproduction can lead to reductions in population-level fitness and the loss of genetic diversity. In contrast, directional dispersal can mitigate the effects of changing environmental conditions by allowing individuals to track optimal conditions. Our results, coupled with previous findings on demography [41], suggest that directional natal dispersal away from warmer and drier climate conditions may explain the local range shift towards higher elevations. Our study presents some of the first evidence of directional natal dispersal in a migratory species.

In a long-term study of female black-throated blue warbler demography, Lewis et al. [41] found that population declines were greater at the trailing edge of the range than at the range core. At both range positions, population density was highest, and trends were most stable, at higher elevations characterized by cooler and wetter climates. Because their study used non-spatial mark-recapture data at six independent sites, they were unable to study dispersal. However, consistent with our results, they found declining per-capita recruitment rates (declining ratio of SY birds on site) at the lowest elevation trailing-edge study plot with the warmest and driest conditions.

Within our study, natal dispersal was more sensitive to climate and occurred over greater distances than adult dispersal. In contrast, adult black-throated blue warblers exhibited strong site fidelity between years as has been documented in the core of the breeding range [9, 31], suggesting that natal dispersal will have more influence on range shifts than adult dispersal. Natal dispersal towards colder and wetter sites may only be possible when populations are below carrying capacity, as high conspecific abundance in the best habitat conditions may preclude young individuals from establishing territories [21]. The documented declines in this population [41] and the apparent infrequency of high elevation nestlings selecting lower elevation territories, suggest that this population is below carrying capacity and low elevation populations will continue to decline.

First year survival is very low for many passerines [42, 44], with apparent survival estimates as low as 5% for some species [63]. While our apparent survival estimate of 10% for first-year birds is similar to estimates of neotropical migrants reported elsewhere [44], we were unable to separate long-distance dispersal from mortality. Our apparent survival estimates are therefore likely lower than actual survival. If these estimates represented true survival, the population would likely decline precipitously. While previous work predicts this species will be locally extirpated from our lower elevation intensive plot by 2030 [41], abundance at high elevation sites is predicted to remain stable. These results suggest that directional natal dispersal of individuals born at warmer and drier sites is maintaining abundance at colder and wetting locations.

The nestling return rates in our study area were substantially higher than those seen in New Hampshire in the core of the breeding range. Holmes et al. [31] reported that only 22 (0.44 %) of >5000 nestlings were known to have returned to the range core between 1986 and 2016. Of these returning birds, the closest return was 300 m from the individual’s natal site, with most birds dispersing more than 500 m, including several dispersal events >2 km from their natal sites. In our study, we observed 24 nestlings returning to the study area, despite having approximately half the sample size of banded nestling black-throated blue warblers and 10 fewer years of data. The high return rate at our study is potentially attributable to the lack of suitable habitat at the trailing-edge where the species is restricted to fragmented high elevation forests.

The availability of cold, high elevation sites in the study area appeared to influence natal dispersal outcomes. Individuals born at the highest elevations had no available colder climate to disperse into within our study area. Even though more than twice the number of nestlings were banded at the highest elevation plot compared to the lower elevation intensive plot, none of these individuals were ever observed establishing territories at the low elevation intensive plot. The lack of movement to the warmer climate at the lower elevation intensive site suggests a pattern of non-random natal dispersal, though more information on long-distance dispersal would help substantiate this hypothesis.

Movements to higher latitude mountains may not represent a significant barrier to black-throated blue warblers and other long-distance migrants that already annually migrate thousands of kilometers [67], but this theory is largely untested. Intraspecific competition or non-thermal abiotic conditions such as habitat quality can prevent individuals from tracking changing climate, even when population movements as a whole trend towards cooler locations [19, 39]. Although the young birds in our study showed flexibility in dispersal distance, it remains unclear if long-distance dispersal to fragmented patches of high quality habitat at higher latitudes is possible. Due to the logistical challenges of studying long-distance movements [34], we restricted our analysis to birds that returned to the study area, but future research should attempt to understand the extent and direction of long-distance dispersal in trailing-edge populations and its role in maintaining population viability under future climate conditions. Advanced monitoring techniques, such as MOTUS and satellite tags applied to first-year birds, may provide a powerful avenue for direct examination of long-distance dispersal.

## Conclusions

For many species, trailing-edge populations act as reservoirs for genetic diversity [1, 24] which can confer higher resistance to environmental change [33, 60, 68]. Previous studies of climate change and dispersal predict an increase in dispersal distances under future climate conditions and more frequent long-distance dispersal events [37, 66]. Our findings suggest that the negative effects of climate change on trailing-edge populations can be mitigated by directional natal dispersal, provided that sufficient connectivity exists between high-quality habitat at the edge and core of the range.

## Data Availability

All data and code to run the analysis in this manuscript are available online: 10.5281/zenodo.7764017.
